# Supplementary material for: Radical Composition and Radical Reaction Kinetics in the Probe-Irradiated XLPE Samples as a Potential Source of Information on Their Aging Degree
Source: Materials (Basel). 2022 Aug 19;15(16):5723. doi: 10.3390/ma15165723 (PMC9414847; doi:10.3390/ma15165723)
Supplement: Supplementary file 1 [file materials-15-05723-s001.zip › materials-1859781-supplementary.pdf]

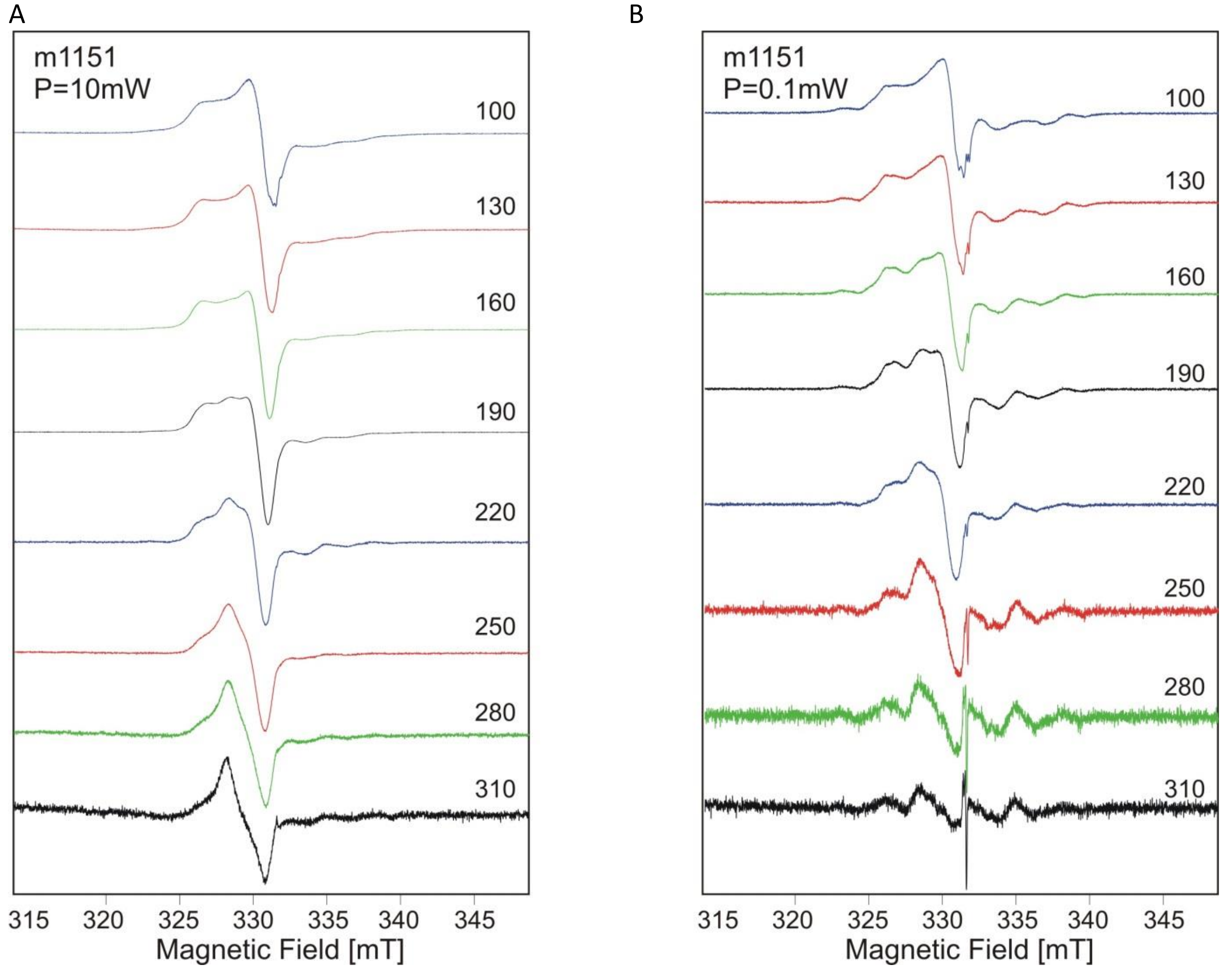

Supplementary Figure S1. The EPR spectra of the AO non-protected XLPE, I-st aging period (low dose rate), probe-irradiated in liquid nitrogen with a dose of 10 kGy, recorded at different temperatures (annealing in the cryostat) in two microwave powers: 10 mW (A) and 0.1 mW (B), respectively.

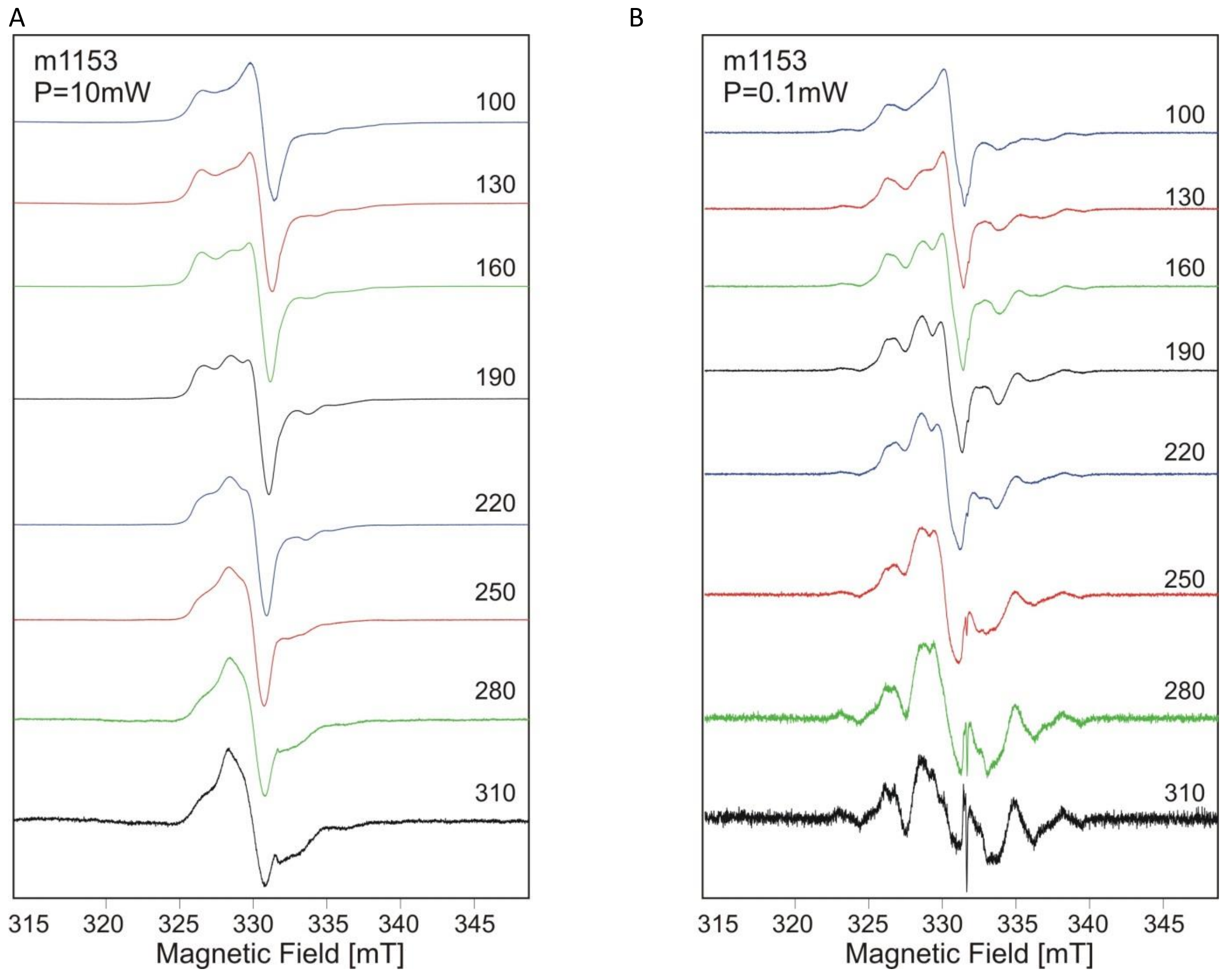

Supplementary Figure S2. The EPR spectra of the AO non-protected XLPE, 3rd aging period (low dose rate), probe-irradiated in liquid nitrogen with a dose of 10 kGy, recorded at different temperatures (annealing in the cryostat) in two microwave powers: 10 mW (A) and 0.1 mW (B), respectively.

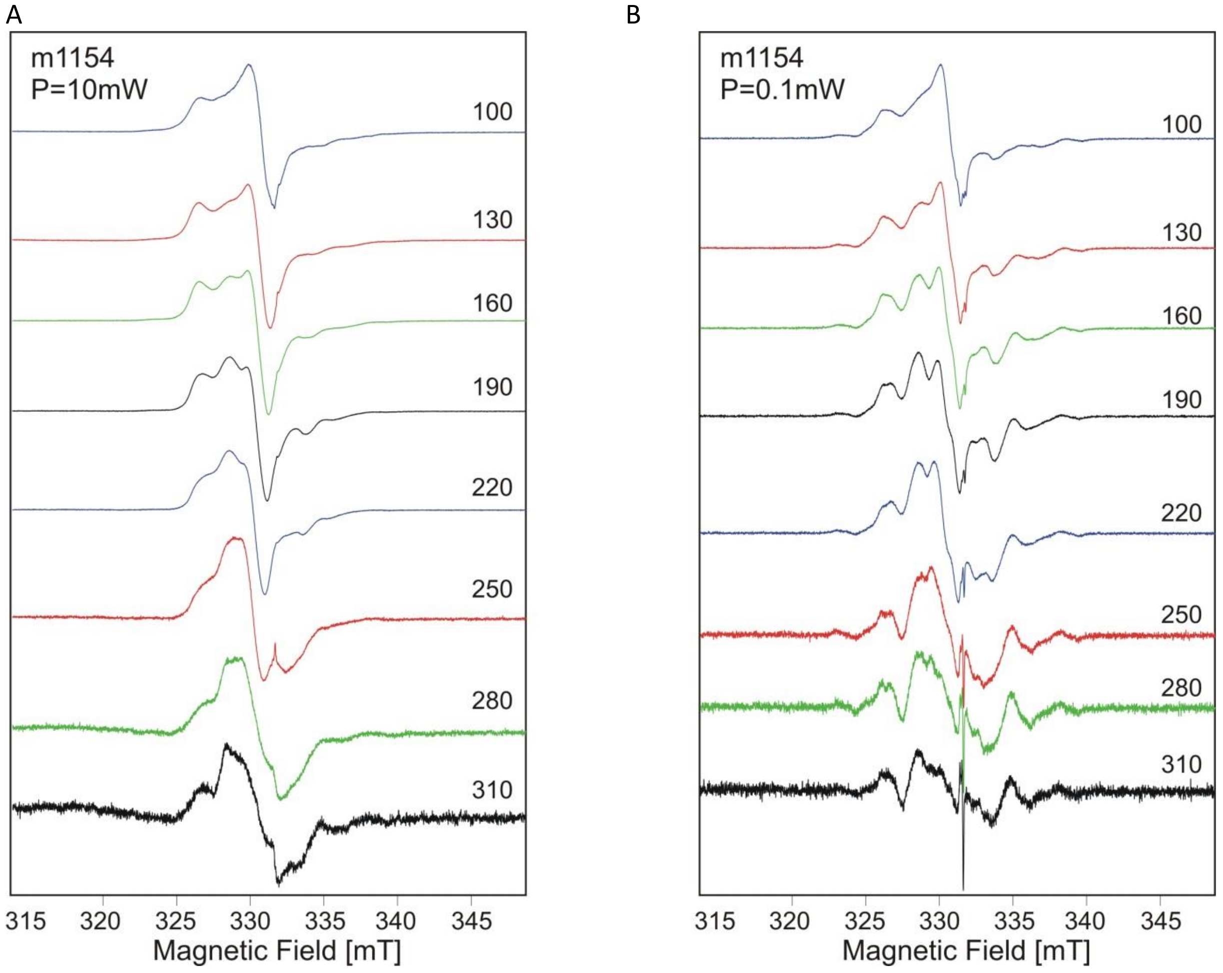

Supplementary Figure S3. The EPR spectra of the AO non-protected XLPE, 4th aging period (low dose rate), probe-irradiated in liquid nitrogen with a dose of 10 kGy, recorded at different temperatures (annealing in the cryostat) in two microwave powers: 10 mW (A) and 0.1 mW (B), respectively.

A

B

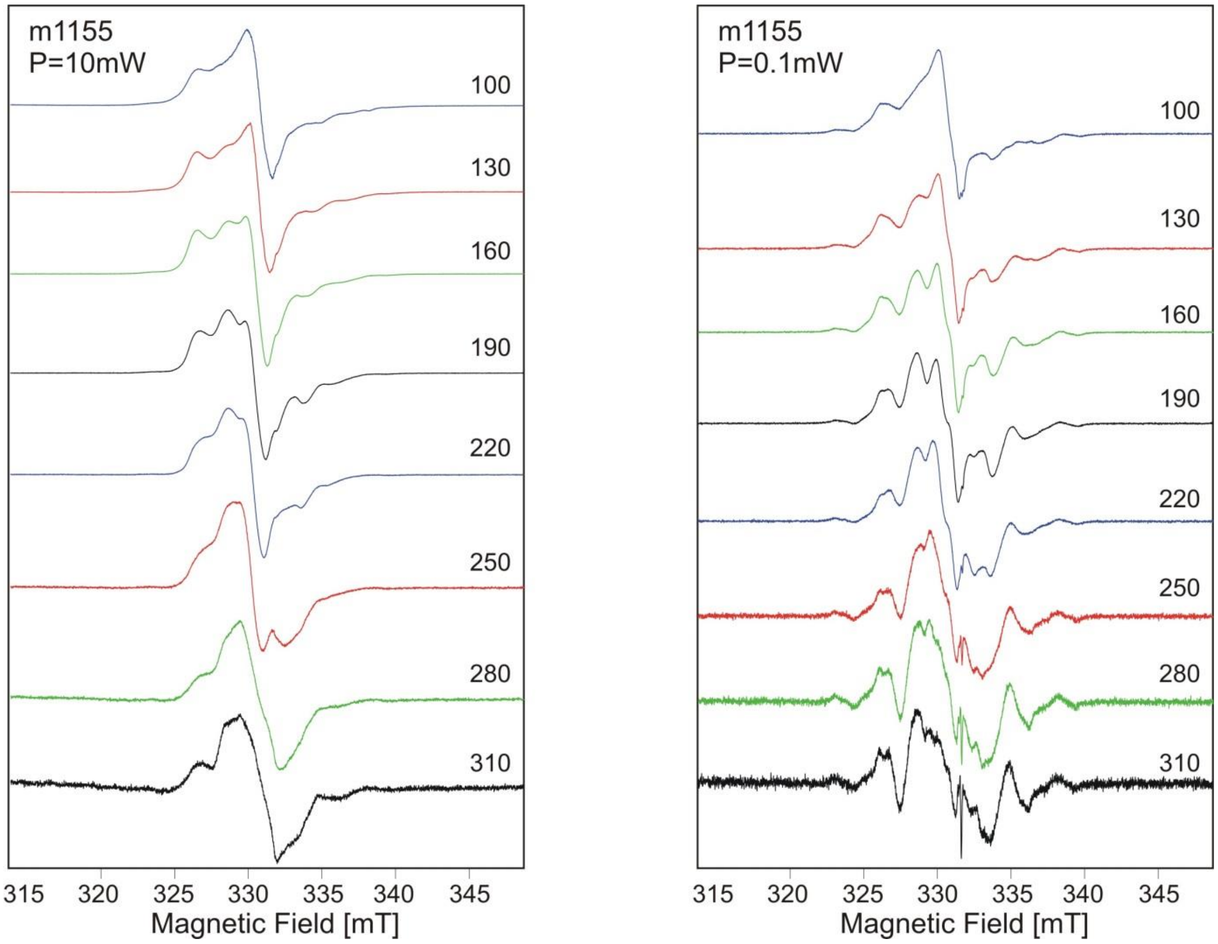

Supplementary Figure S4. The EPR spectra of the AO non-protected XLPE, 5th aging period (low dose rate), probe-irradiated in liquid nitrogen with a dose of 10 kGy, recorded at different temperatures (annealing in the cryostat) in two microwave powers: 10 mW (A) and 0.1 mW (B), respectively.

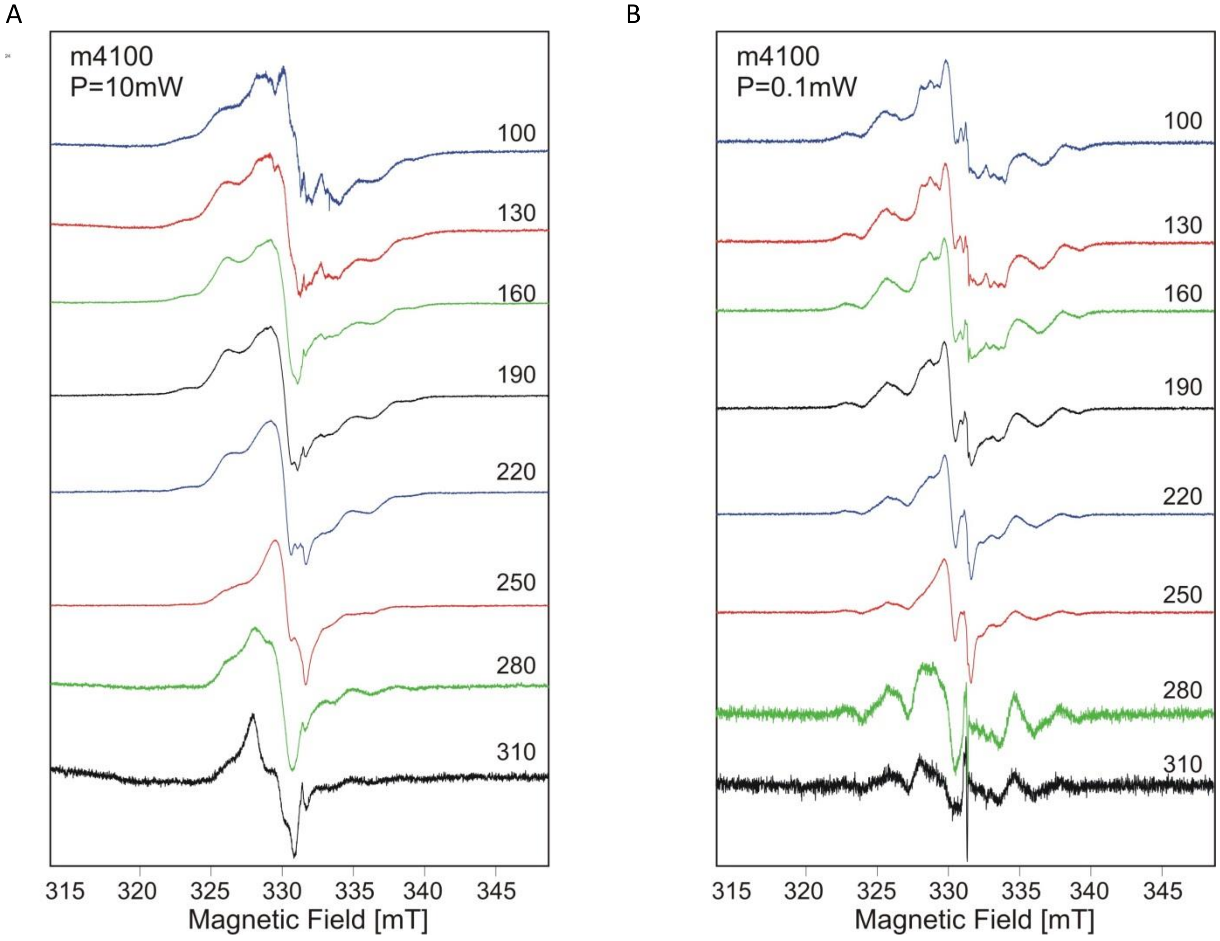

Supplementary Figure S5. The EPR spectra of the AO-protected XLPE, non-aged, probe-irradiated in liquid nitrogen with a dose of 10 kGy, recorded at different temperatures (annealing in the cryostat) in two microwave powers: 10 mW (A) and 0.1 mW (B), respectively.

A B

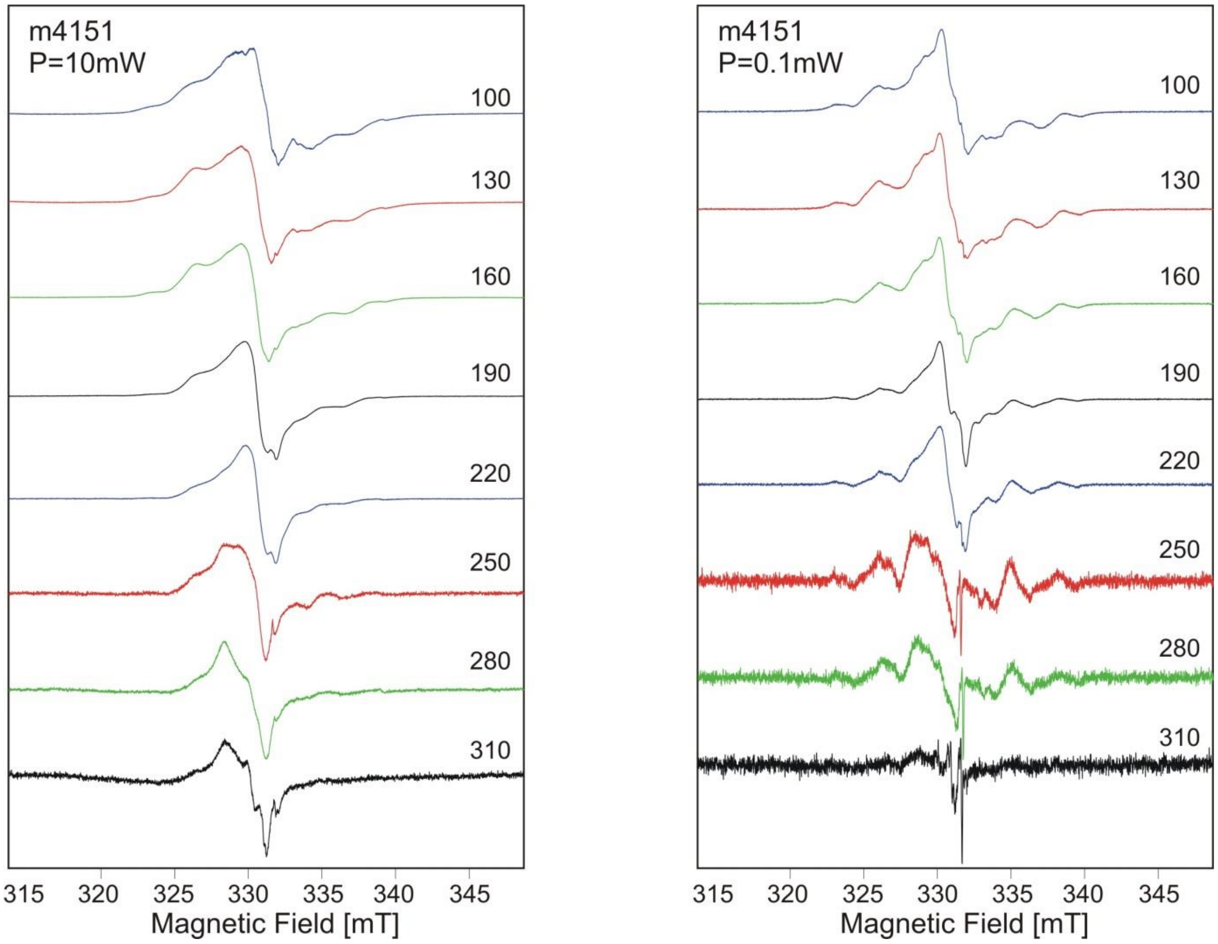

Supplementary Figure S6. The EPR spectra of the AO-protected XLPE, I-st aging period (low dose rate), probe-irradiated in liquid nitrogen with a dose of 10 kGy, recorded at different temperatures (annealing in the cryostat) in two microwave powers: 10 mW (A) and 0.1 mW (B), respectively.

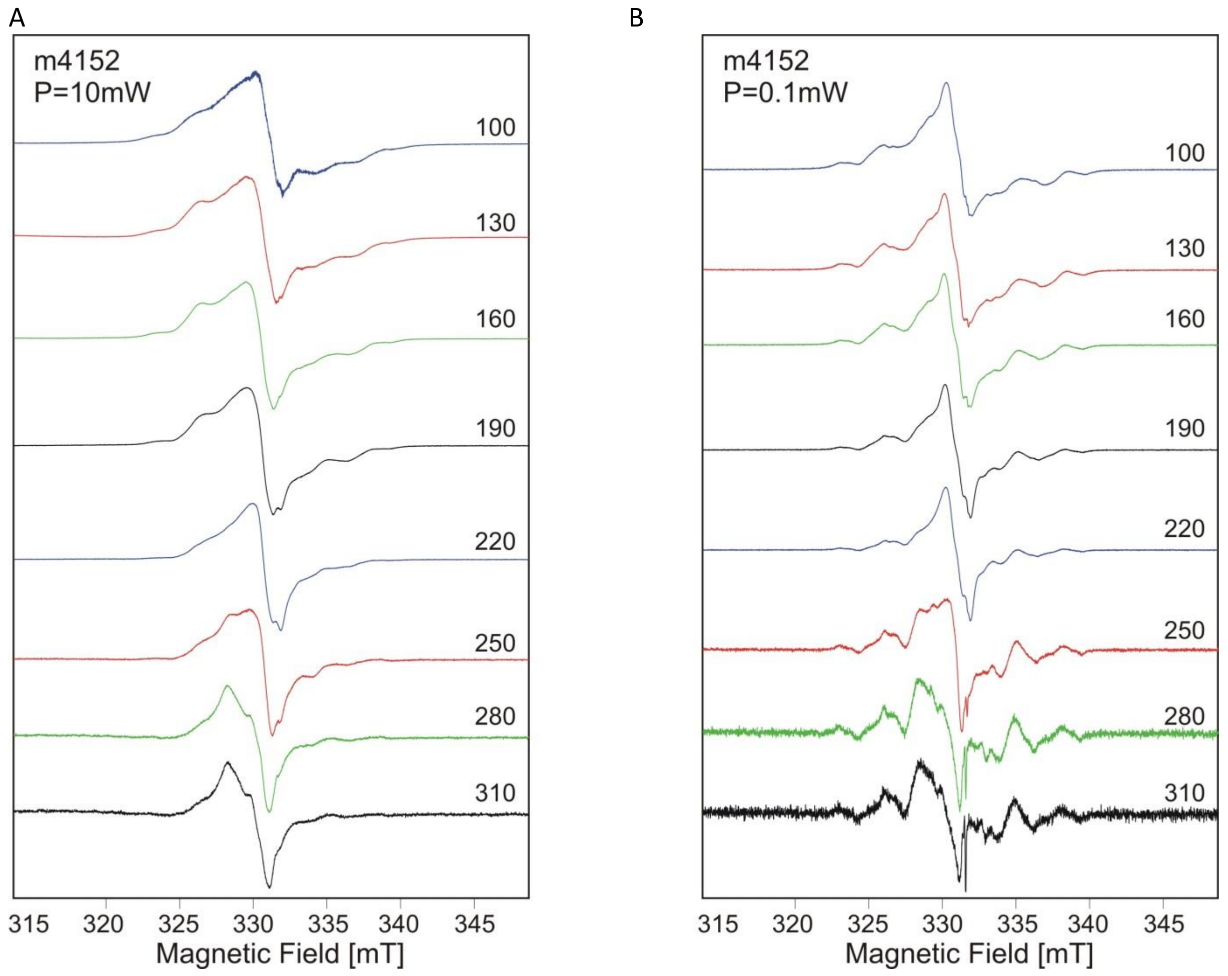

Supplementary Figure S7. The EPR spectra of the AO-protected XLPE, 2nd aging period (low dose rate), probe-irradiated in liquid nitrogen with a dose of 10 kGy, recorded at different temperatures (annealing in the cryostat) in two microwave powers: 10 mW (A) and 0.1 mW (B), respectively.

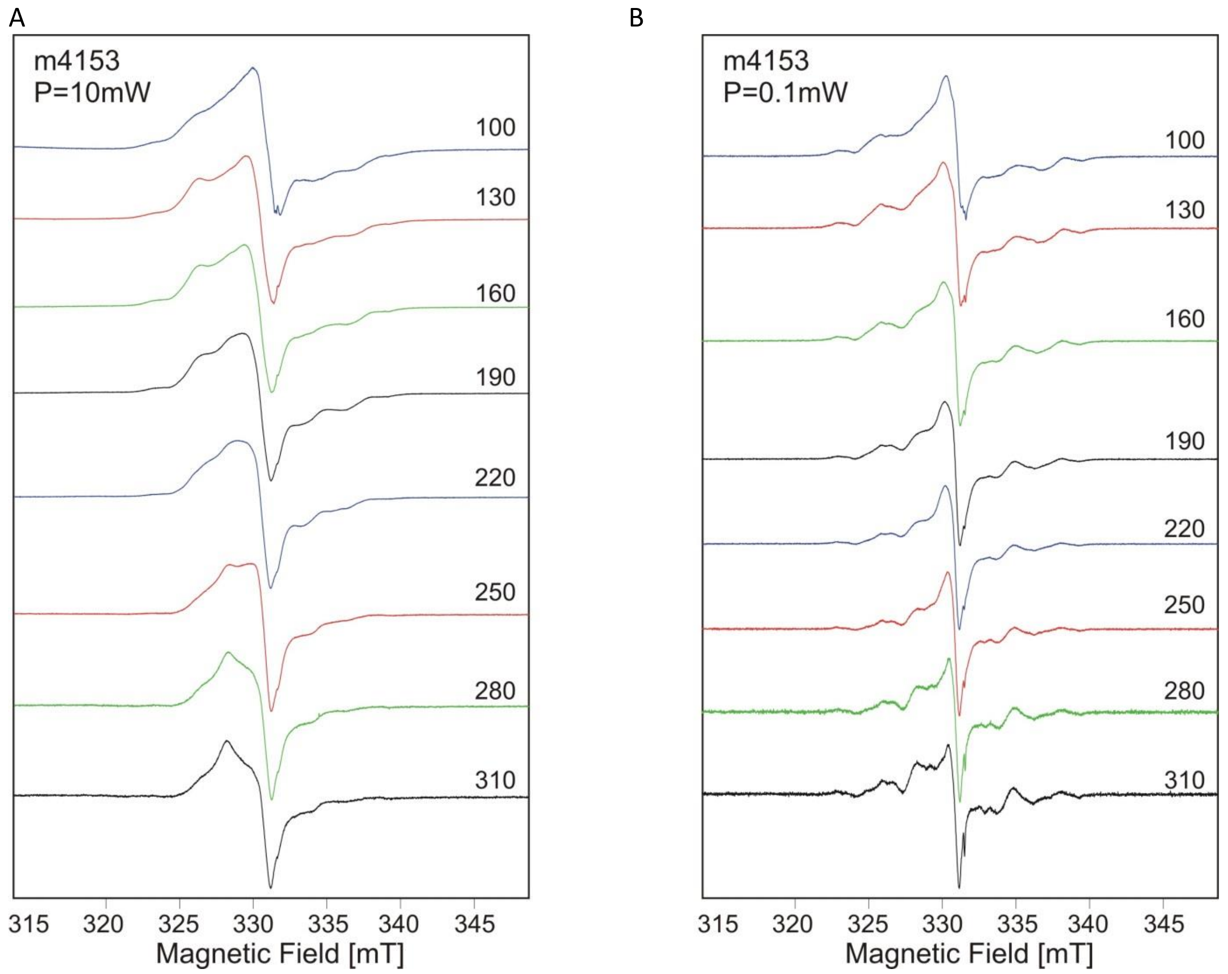

Supplementary Figure S8. The EPR spectra of the AO-protected XLPE, 3rd aging period (low dose rate), probe-irradiated in liquid nitrogen with a dose of 10 kGy, recorded at different temperatures (annealing in the cryostat) in two microwave powers: 10 mW (A) and 0.1 mW (B), respectively.

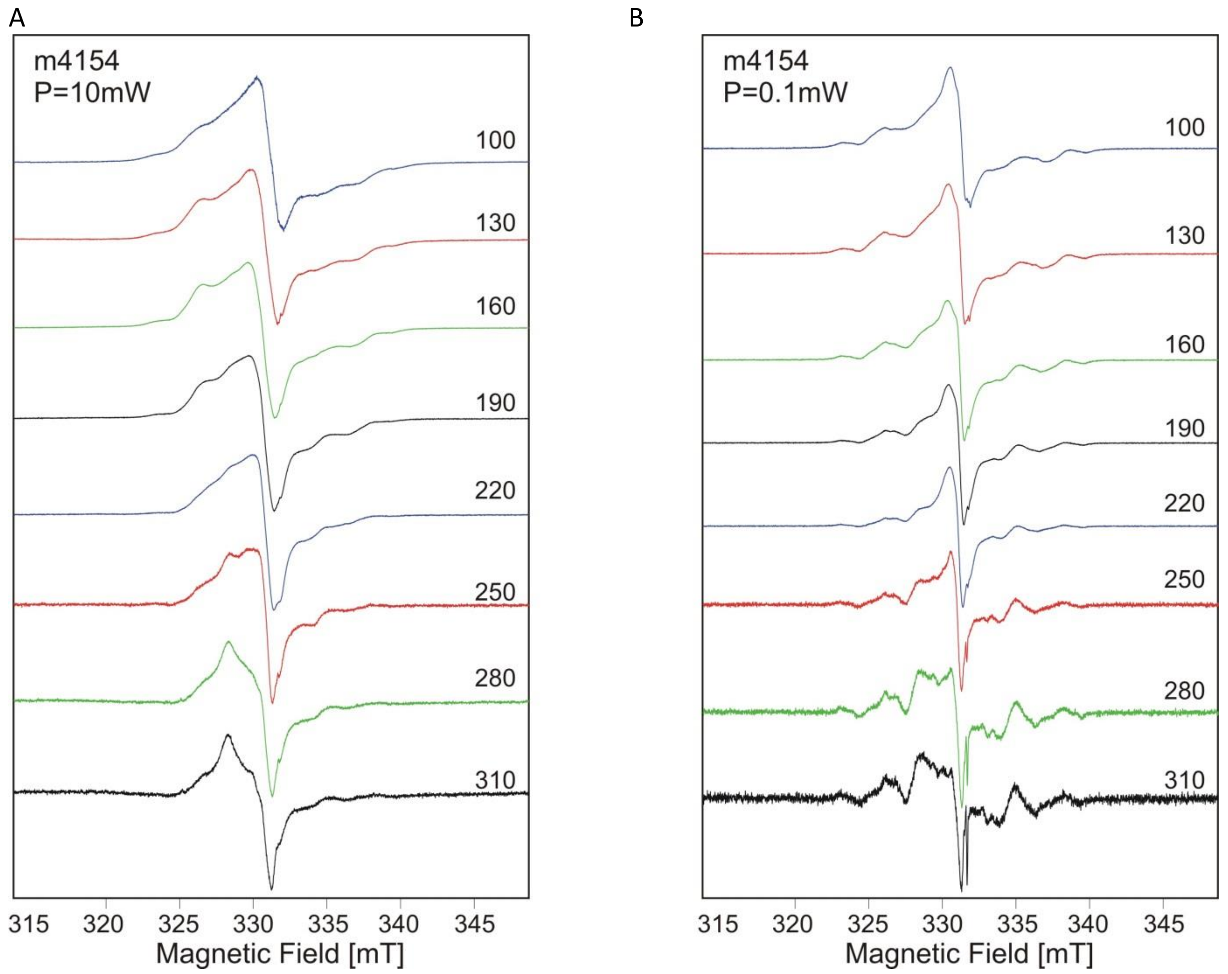

Supplementary Figure S9. The EPR spectra of the AO-protected XLPE, 4th aging period (low dose rate), probe-irradiated in liquid nitrogen with a dose of 10 kGy, recorded at different temperatures (annealing in the cryostat) in two microwave powers: 10 mW (A) and 0.1 mW (B), respectively.

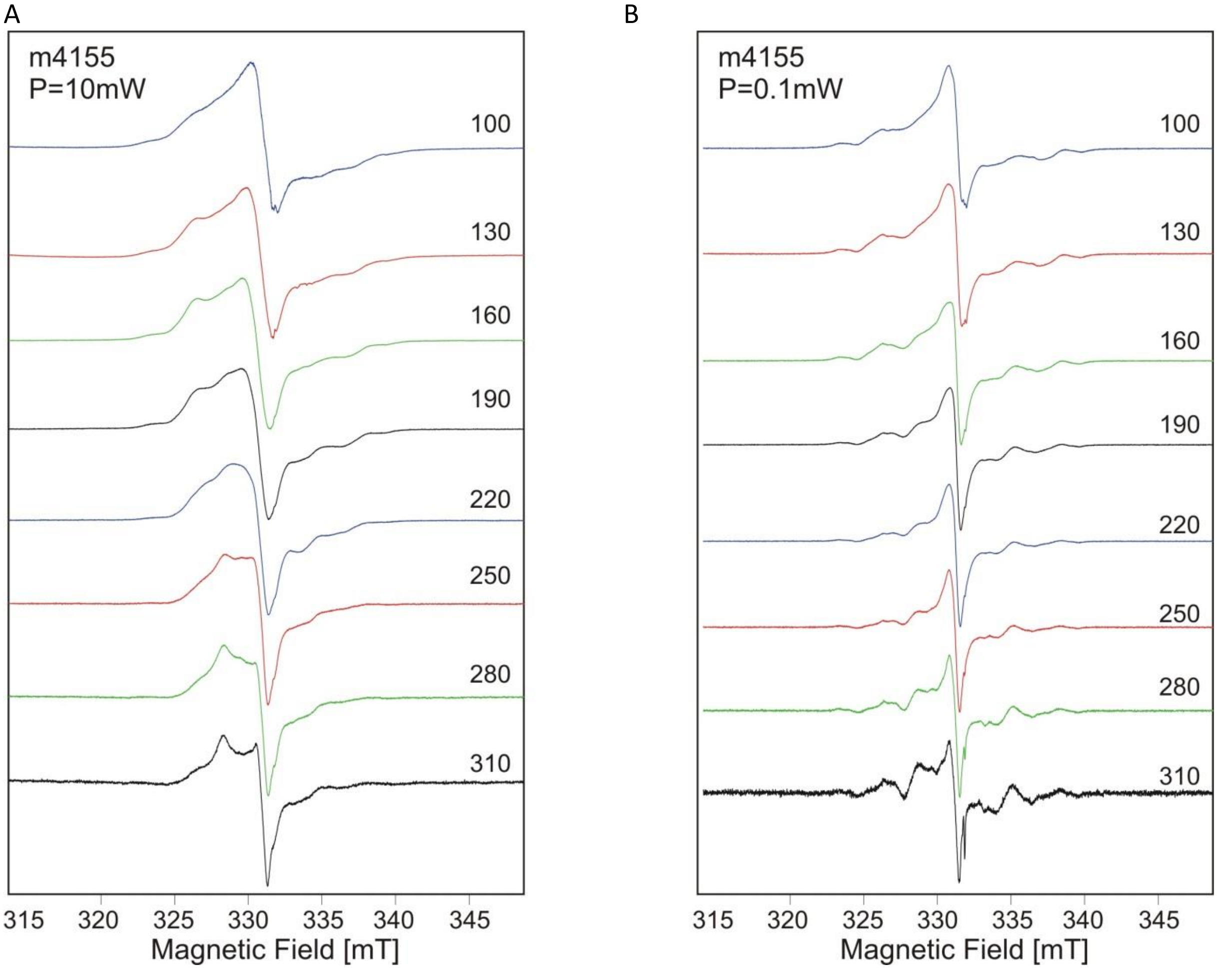

Supplementary Figure S10. The EPR spectra of the AO-protected XLPE, 5th aging period (low dose rate), probe-irradiated in liquid nitrogen with a dose of 10 kGy, recorded at different temperatures (annealing in the cryostat) in two microwave powers: 10 mW (A) and 0.1 mW (B), respectively.

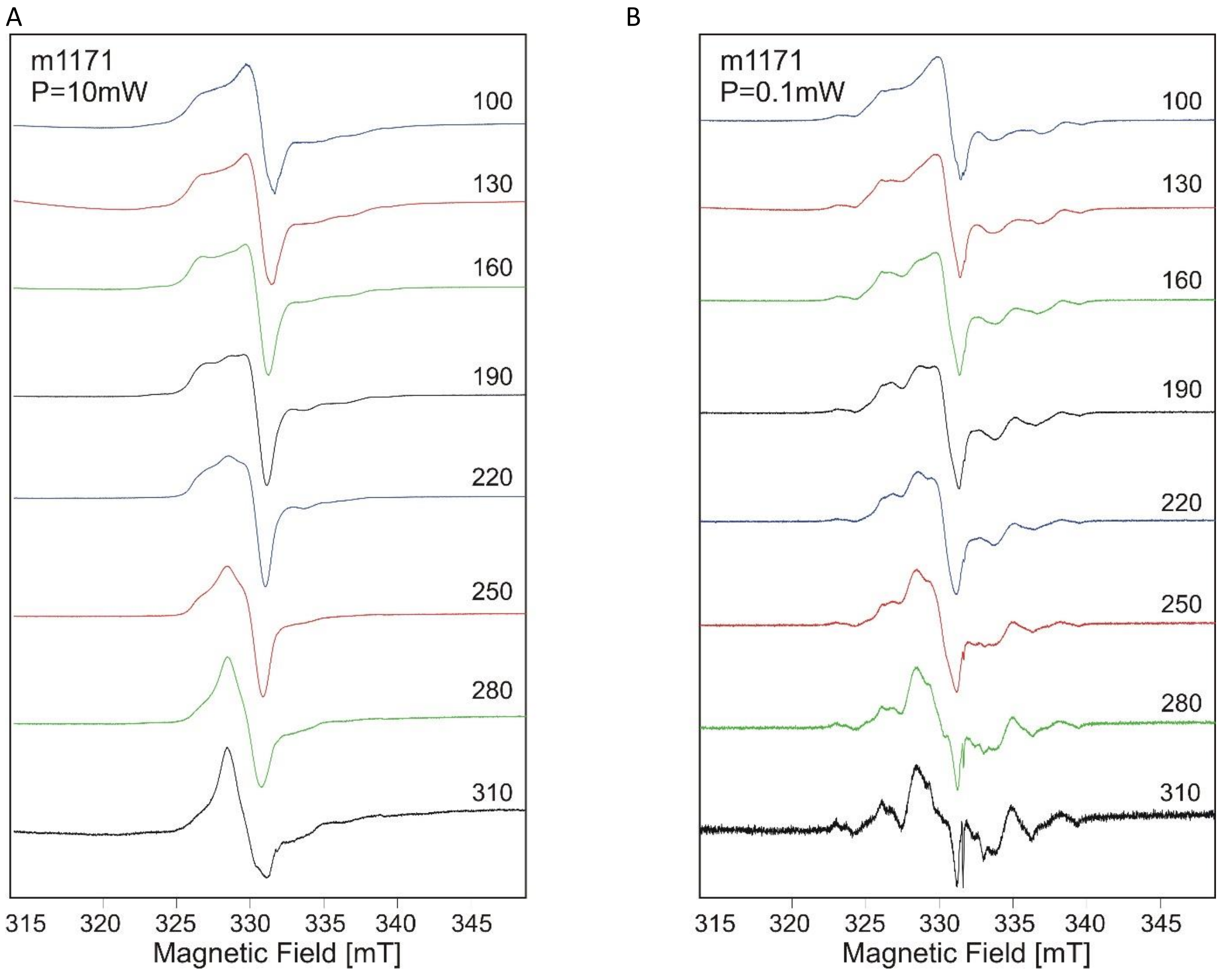

Supplementary Figure S11. The EPR spectra of the AO non-protected XLPE, I-st aging period (high dose rate), probe-irradiated in liquid nitrogen with a dose of 10 kGy, recorded at different temperatures (annealing in the cryostat) in two microwave powers: 10 mW (A) and 0.1 mW (B), respectively.

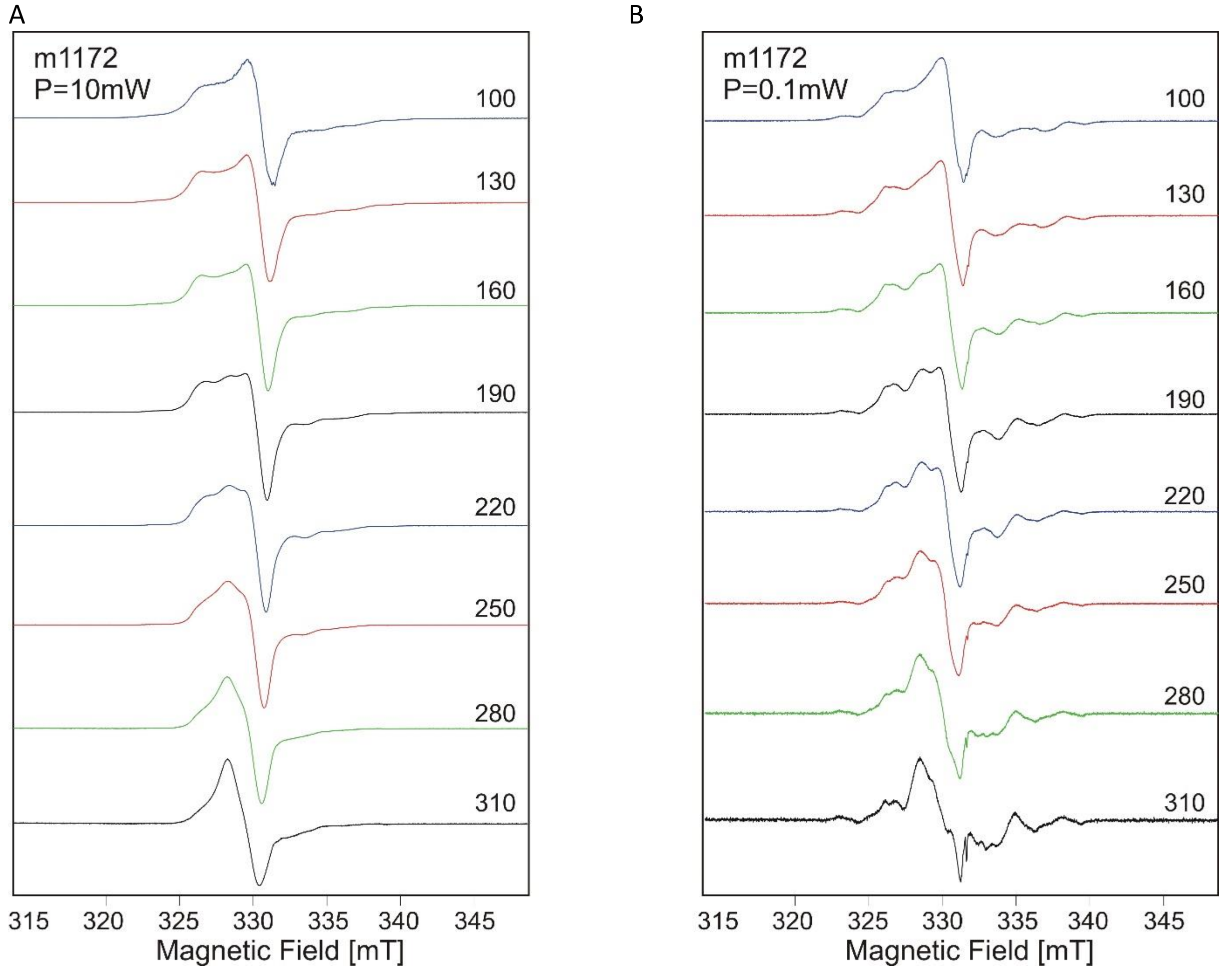

Supplementary Figure S12. The EPR spectra of the AO non-protected XLPE, 2nd aging period (high dose rate), probe-irradiated in liquid nitrogen with a dose of 10 kGy, recorded at different temperatures (annealing in the cryostat) in two microwave powers: 10 mW (A) and 0.1 mW (B), respectively.

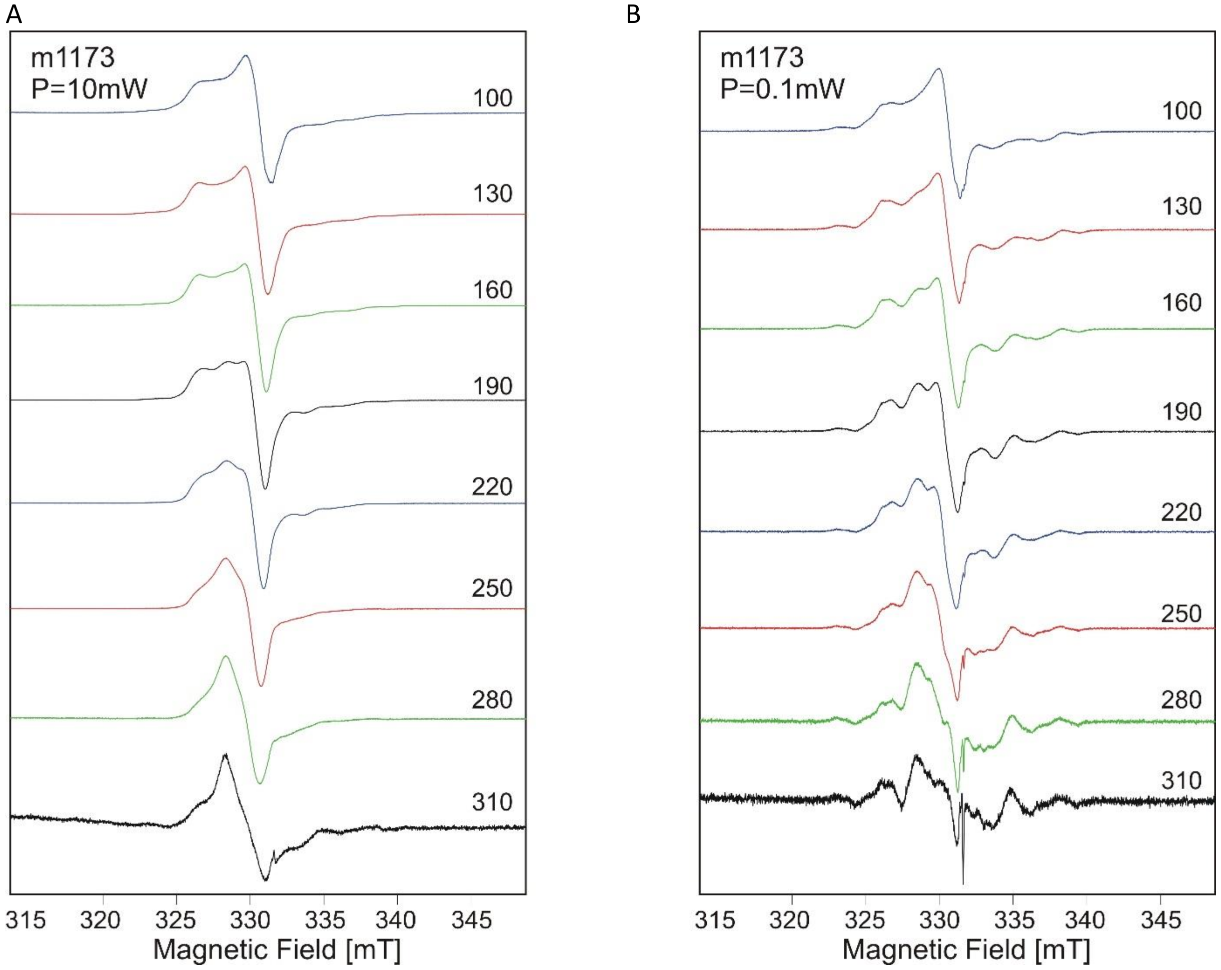

Supplementary Figure S13. The EPR spectra of the AO non-protected XLPE, 3rd aging period (high dose rate), probe-irradiated in liquid nitrogen with a dose of 10 kGy, recorded at different temperatures (annealing in the cryostat) in two microwave powers: 10 mW (A) and 0.1 mW (B), respectively.

A

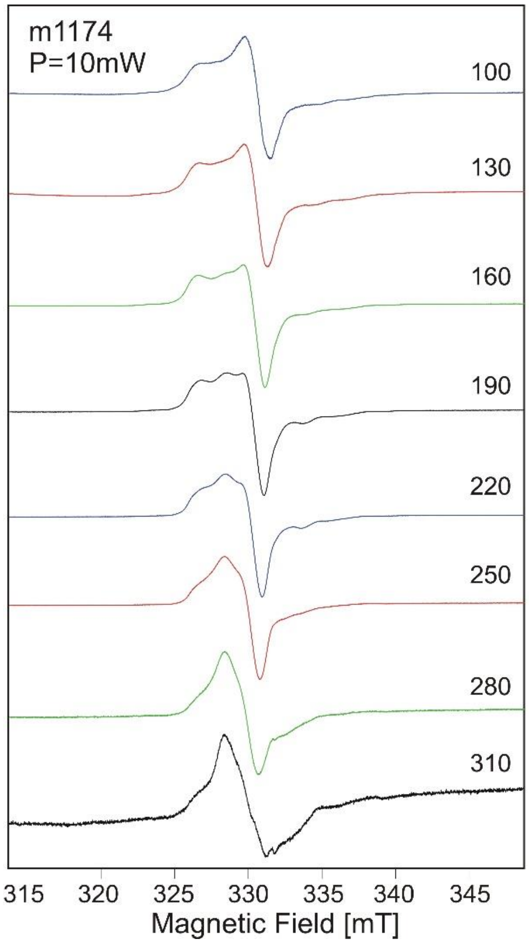

B

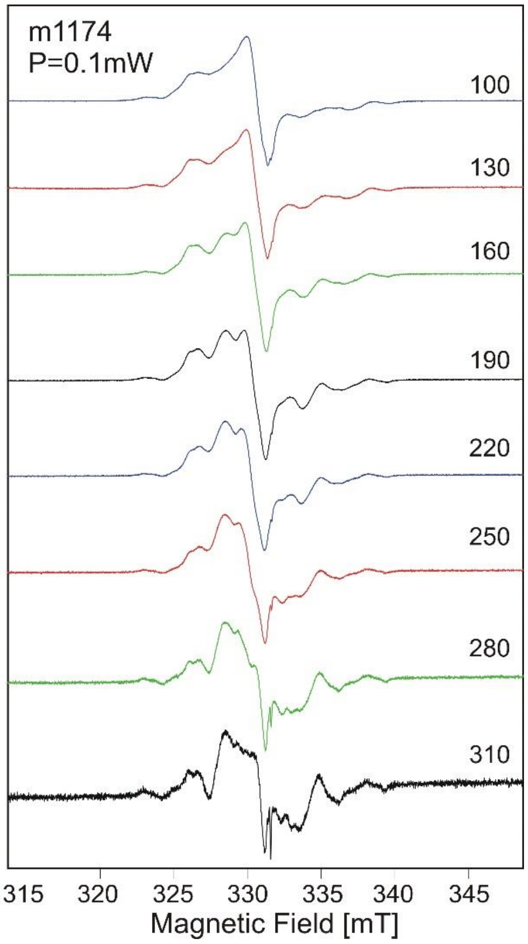

Supplementary Figure S14. The EPR spectra of the AO non-protected XLPE, 4th aging period (high dose rate), probe-irradiated in liquid nitrogen with a dose of 10 kGy, recorded at different temperatures (annealing in the cryostat) in two microwave powers: 10 mW (A) and 0.1 mW (B), respectively.

A

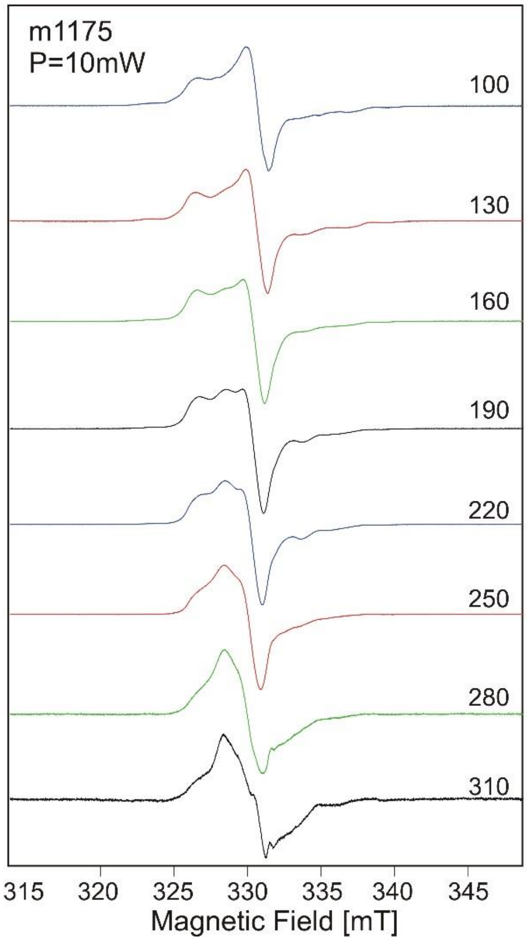

B

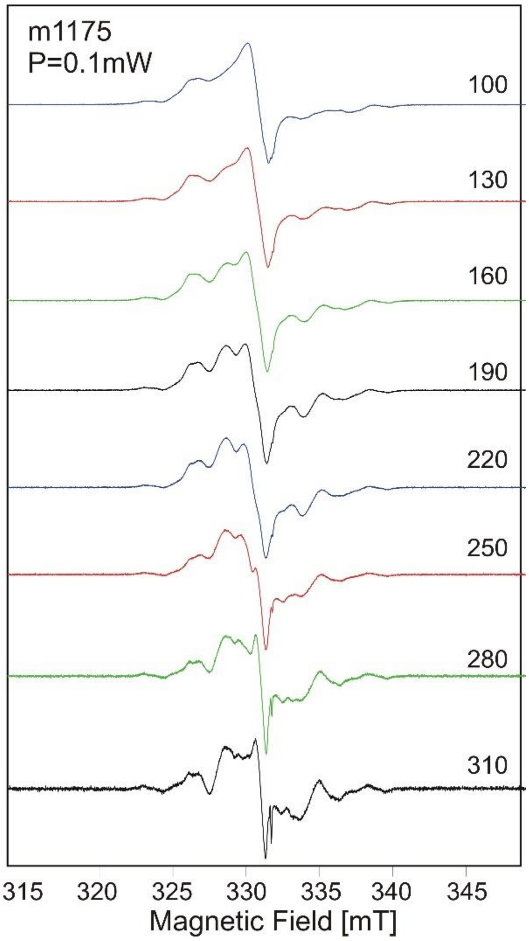

Supplementary Figure S15. The EPR spectra of the AO non-protected XLPE, 5th aging period (high dose rate), probe-irradiated in liquid nitrogen with a dose of 10 kGy, recorded at different temperatures (annealing in the cryostat) in two microwave powers: 10 mW (A) and 0.1 mW (B), respectively.

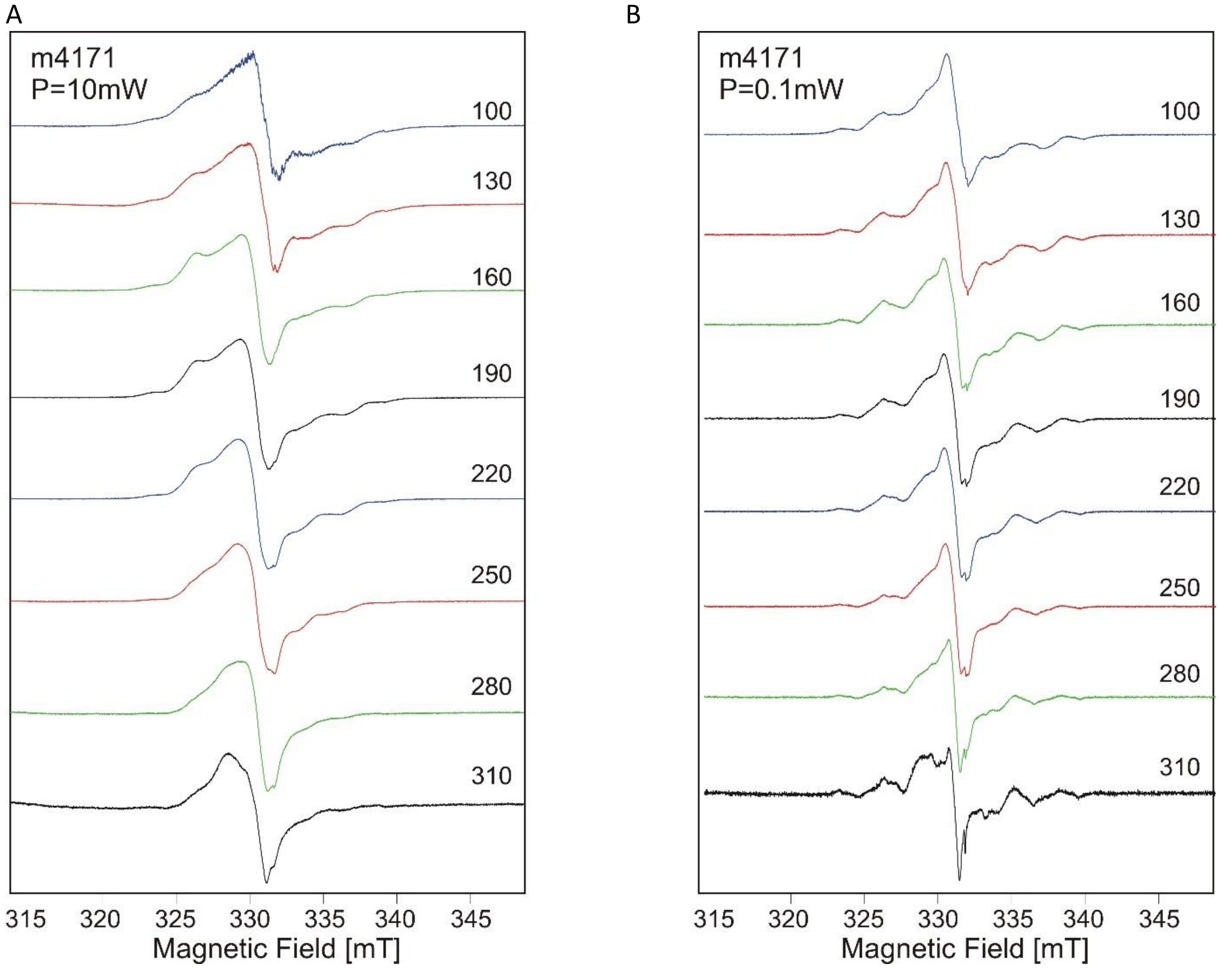

Supplementary Figure S16. The EPR spectra of the AO-protected XLPE, I-st aging period (high dose rate), probe-irradiated in liquid nitrogen with a dose of 10 kGy, recorded at different temperatures (annealing in the cryostat) in two microwave powers: 10 mW (A) and 0.1 mW (B), respectively.

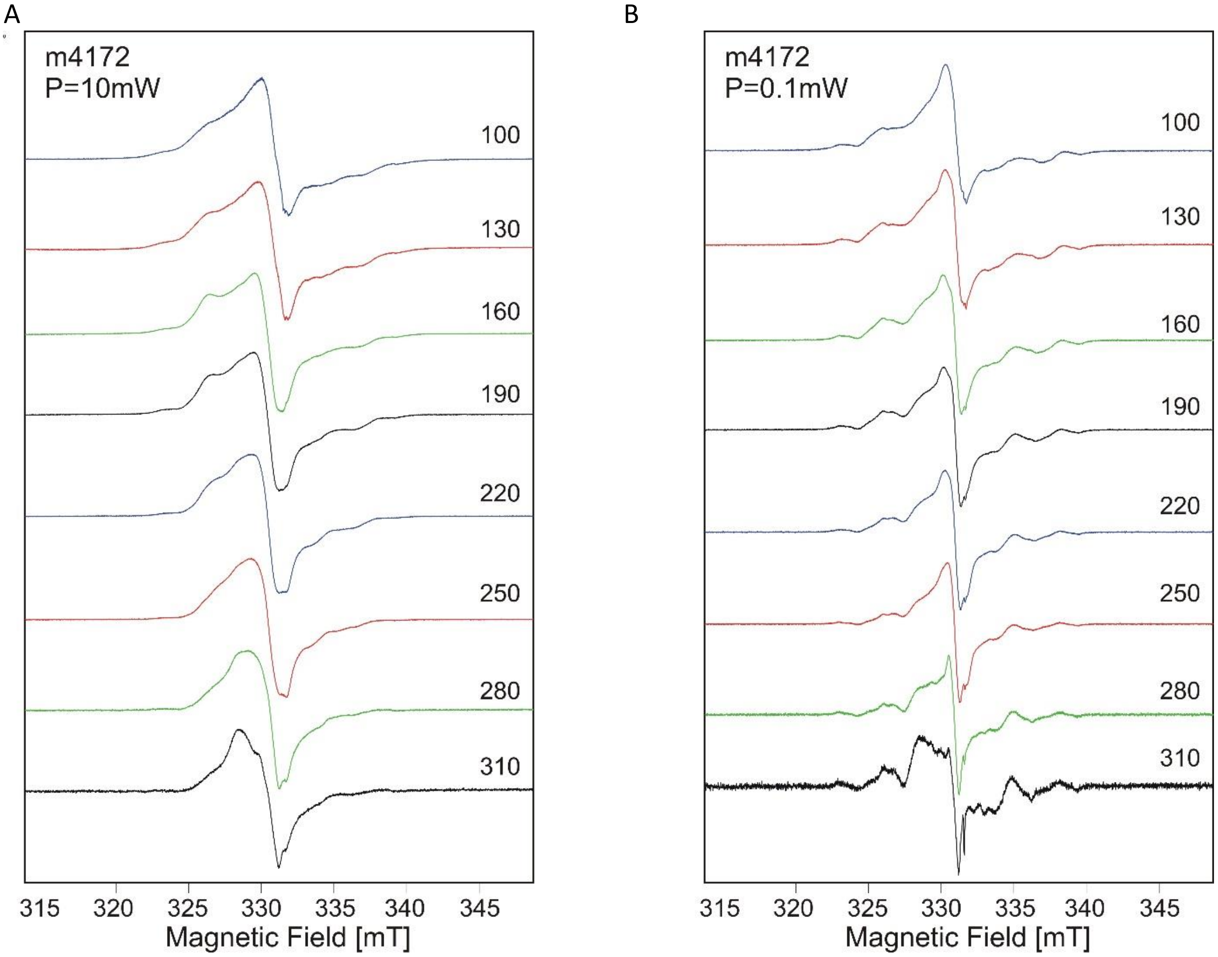

Supplementary Figure S17. The EPR spectra of the AO-protected XLPE, 2nd aging period (high dose rate), probe-irradiated in liquid nitrogen with a dose of 10 kGy, recorded at different temperatures (annealing in the cryostat) in two microwave powers: 10 mW (A) and 0.1 mW (B), respectively.

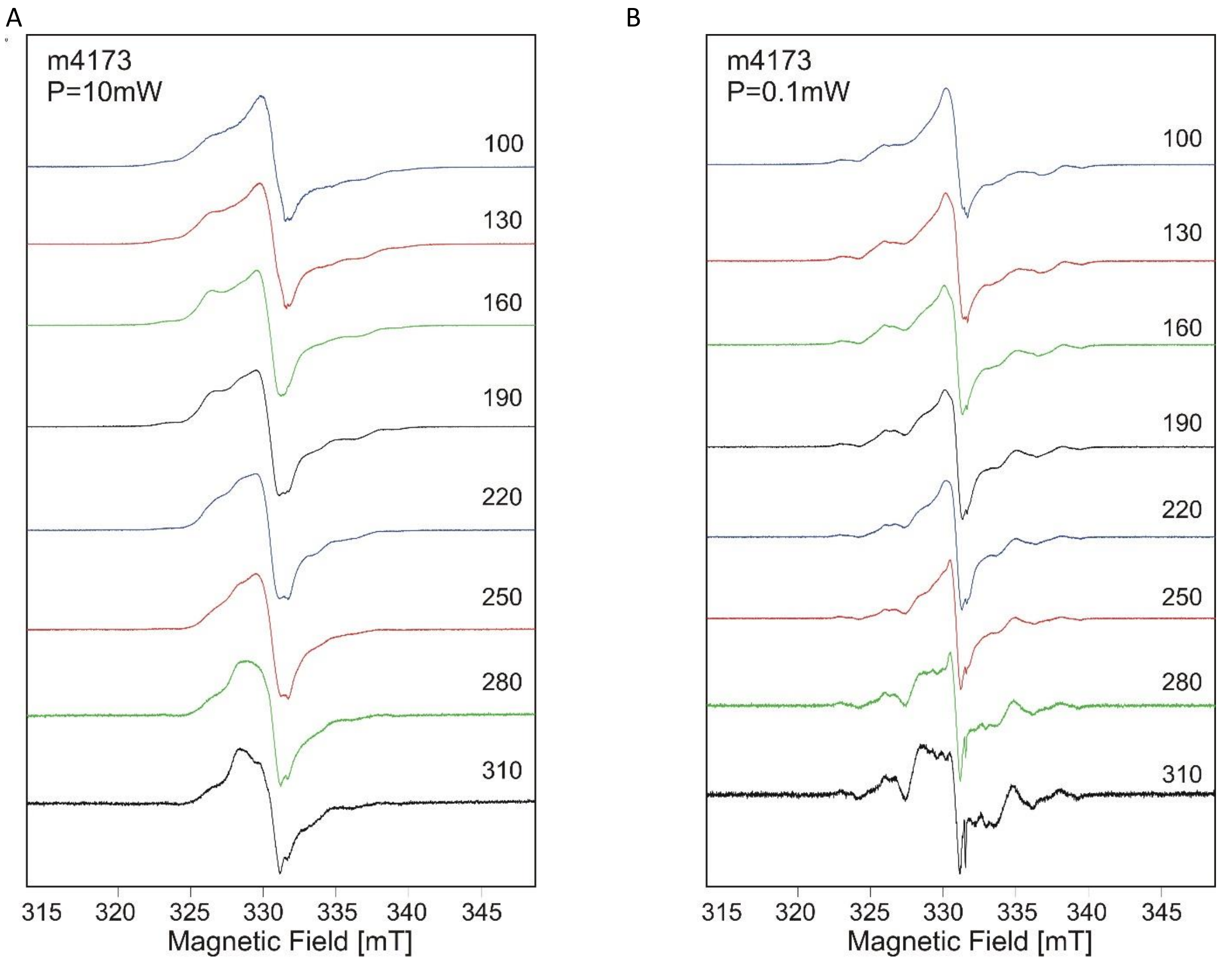

Supplementary Figure S18. The EPR spectra of the AO-protected XLPE, 3rd aging period (high dose rate), probe-irradiated in liquid nitrogen with a dose of 10 kGy, recorded at different temperatures (annealing in the cryostat) in two microwave powers: 10 mW (A) and 0.1 mW (B), respectively.

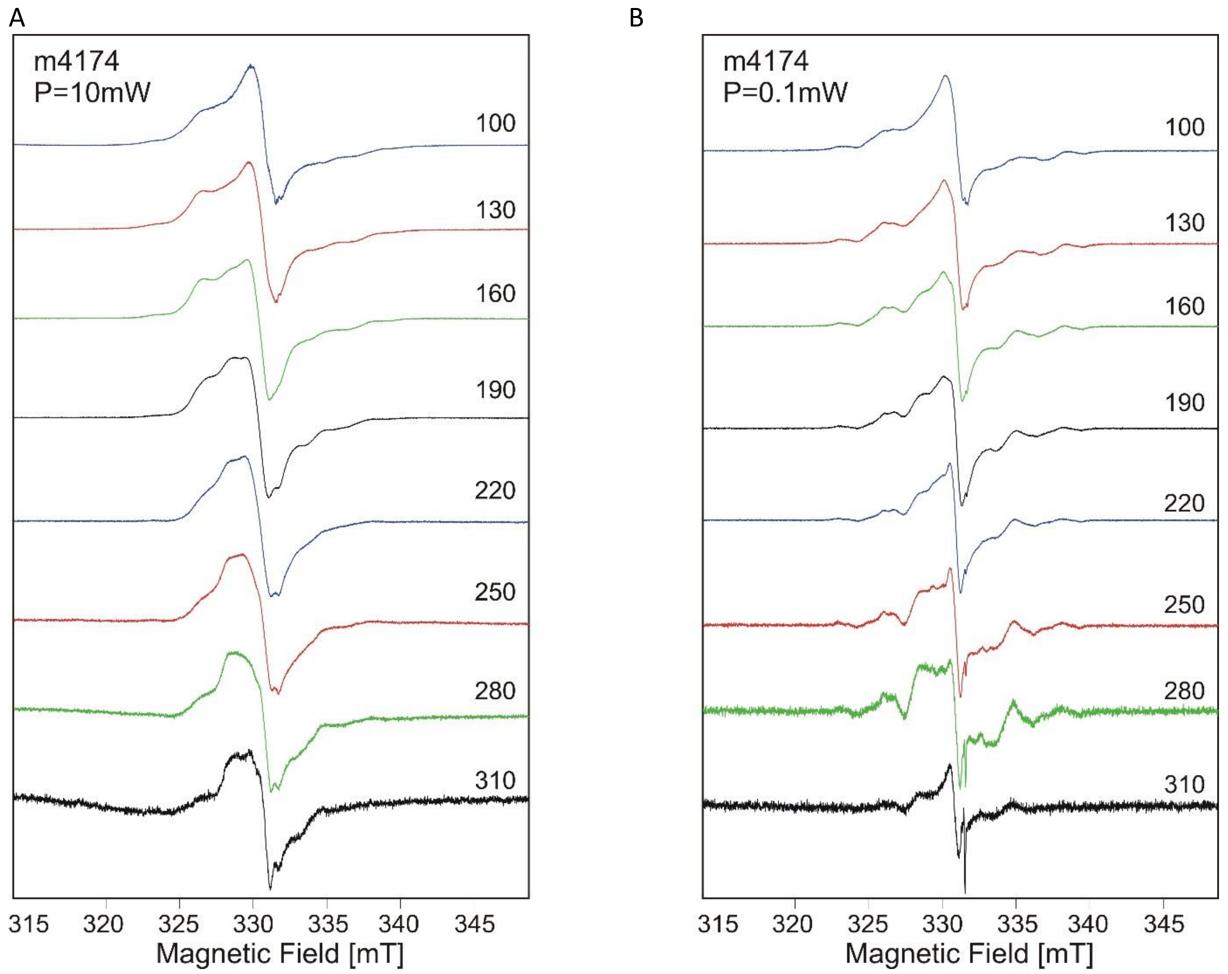

Supplementary Figure S19. The EPR spectra of the AO-protected XLPE, 4th aging period (high dose rate), probe-irradiated in liquid nitrogen with a dose of 10 kGy, recorded at different temperatures (annealing in the cryostat) in two microwave powers: 10 mW (A) and 0.1 mW (B), respectively.

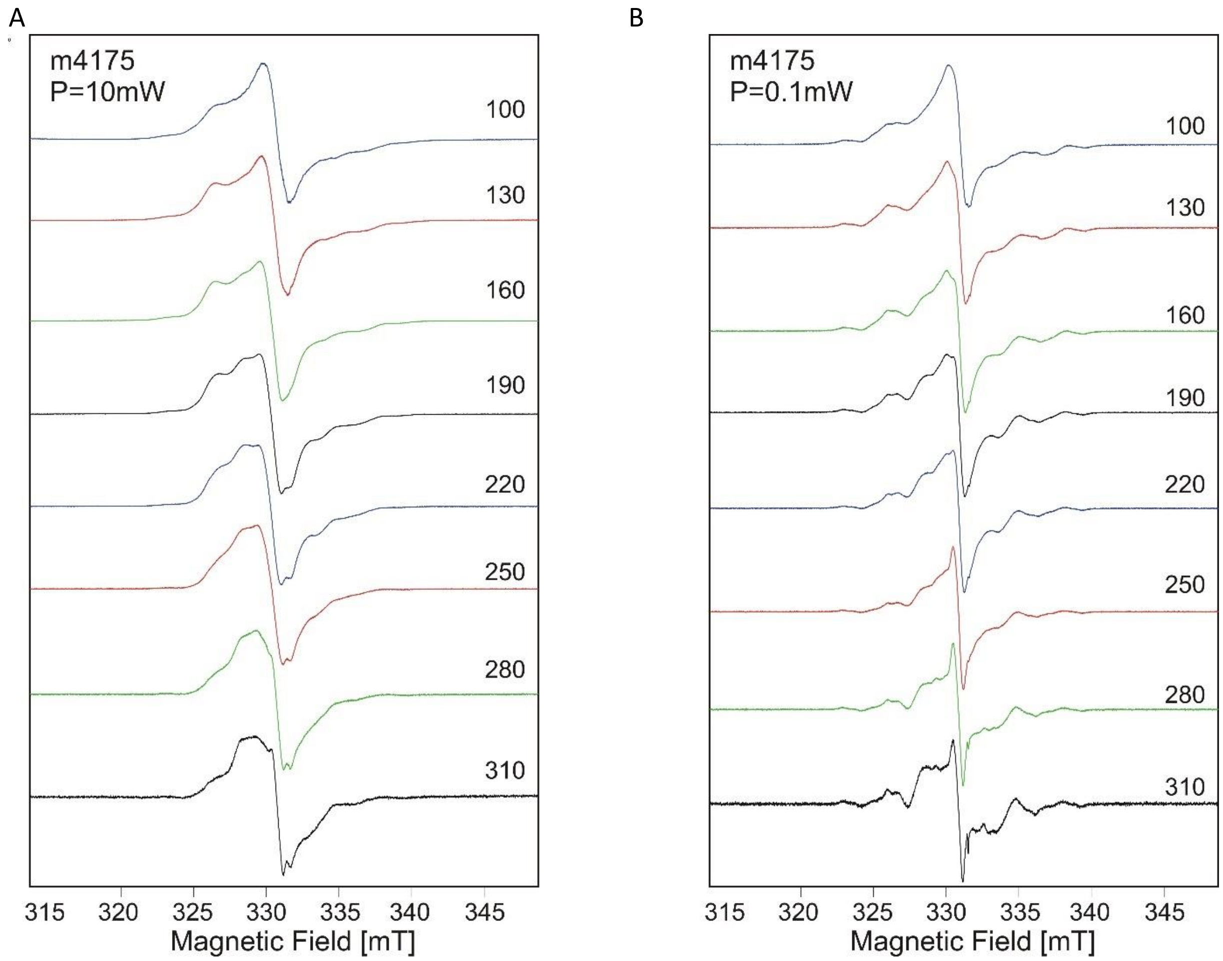

Supplementary Figure S20. The EPR spectra of the AO-protected XLPE, 5th aging period (high dose rate), probe-irradiated in liquid nitrogen with a dose of 10 kGy, recorded at different temperatures (annealing in the cryostat) in two microwave powers: 10 mW (A) and 0.1 mW (B), respectively.
